# Supplementary material for: Dynalign II: common secondary structure prediction for RNA homologs with domain insertions
Source: Nucleic Acids Res. 2014 Nov 21;42(22):13939–48. doi: 10.1093/nar/gku1172 (PMC4267632; doi:10.1093/nar/gku1172)
Supplement: SUPPLEMENTARY DATA [file supp_gku1172_nar-02021-z-2014-File012.zip › manual/RNA_class/html/functions_0x66.html]

RNAstructure Classes: Class Members

- Main Page
- Classes
- Files

- Class List
- Class Hierarchy
- Class Members

- All
- Functions
- Variables
- Typedefs

- a
- b
- c
- d
- e
- f
- g
- h
- i
- l
- m
- n
- o
- p
- q
- r
- s
- t
- v
- w
- ~

Here is a list of all class members with links to the classes they belong to:

### - f -

- fce
  : RNA- FileReader()
    : RNA- FoldBimolecular()
      : HybridRNA- FoldDuplex()
        : HybridRNA- FoldSingleStrand()
          : RNA- forbidunimolecular
            : HybridRNA- forcealign
              : Dynalign\_object- ForceAlignment()
                : Dynalign\_object- ForceDoubleStranded()
                  : RNA- ForceFMNCleavage()
                    : RNA- ForceMaximumPairingDistance()
                      : RNA- ForceModification()
                        : RNA- ForcePair()
                          : RNA- ForceProhibitPair()
                            : RNA- ForceSingleStranded()
                              : RNA

---

Generated on Tue Jul 30 10:52:29 2013 for RNAstructure Classes by 
 1.5.7.1
